# Registered Nurses' Digital Client Work and Associating Factors: A Cross‐Sectional Study

**DOI:** 10.1111/jan.16485

**Published:** 2024-09-28

**Authors:** Emma Kainiemi, Anu‐Marja Kaihlanen, Lotta Virtanen, Tuulikki Vehko, Tarja Heponiemi

**Affiliations:** ^1^ Finnish Institute for Health and Welfare Helsinki Finland

**Keywords:** digital client work, digital services, registered nurses, secured messaging, telehealth, video consultations

## Abstract

**Aims:**

To describe the frequency of digital client work among Finnish registered nurses, including video consultations, secured messaging and digital promotion of care without direct contact with the client. In addition, the study examines the association between various factors related to nurses' characteristics and work environment with digital client work and its frequency.

**Design:**

A cross‐sectional survey study.

**Methods:**

A total of 2970 nurses responded to a nationwide survey in spring 2023. Descriptive statistics were used to characterise the frequency of different types of digital client work. Binary logistic regression analyses were used to examine the associations.

**Results:**

One‐third of the respondents reported digital client work during the last 6 months. The majority had worked digitally with their clients daily or weekly. Secured messaging was the most frequently used type of digital client work, whereas video consultations were less frequent. Nurses working in acute care, home‐based care or other environments worked more frequently digitally with their clients than those working in inpatient care. Nurses with higher digital dedication and collegial support had greater odds of digital client work than those with lower dedication. Among those who reported frequent digital client work, lower skills in information security were observed.

**Conclusions:**

Given the significant variation in the frequency of digital client work among nurses across different environments, assessing broader digitalisation adoption opportunities is essential. Organisations must ensure that nurses have sufficient skills for secure handling of client data, and efforts should be made in creating motivational and supportive work environments to facilitate digital client work.

**Implications:**

By understanding the factors influencing nurses' digital client work, organisations can create stronger structures to support their work. Enhancing digital service availability across different healthcare settings would offer clients more care options, thereby potentially improving their access to healthcare.

**Impact:**

This research addresses a knowledge gap regarding the current extent of nurses' digital client work in various healthcare environments and explores potential influencing factors. As governments aim to significantly expand the provision of digital healthcare services, understanding the variation in nurses' digital client work is crucial. This information can guide targeted interventions, such as continuous education, and organisational and collegial support, facilitating dedication to use digital technologies and ensuring secure and impactful advancements in digital healthcare. Our research will benefit healthcare organisations, decision‐makers, nursing professionals and educational institutions.

**Reporting Method:**

Our study adheres to the relevant EQUATOR guidelines and follows the STROBE checklist for cross‐sectional studies.

**Patient or Public Contribution:**

No patient or public contribution.

**Summary**

- What does this paper contribute to the wider global clinical community?
  ○ The study provides valuable insights into the nurses' digital client work across diverse working environments, illuminating the current situation and identifying environments where the utilisation is currently limited, thereby expanding care alternatives for clients.
  ○ The study highlights the importance of fostering a motivational and supportive working environment and providing educational resources for nurses to effectively navigate digital environments, enabling them to ultimately deliver enhanced digital services to their clients.

## 1 Introduction

Great expectations have been set internationally for digital services imperative to address the challenges within the healthcare sector (Organisation for Economic Cooperation and Development [OECD] 2023; World Health Organisation 2021). For example, EU policies consistently underscore the importance of digital solutions, emphasising their potential to enhance care integration and provide more person‐centred and efficient healthcare, ultimately resulting in resource savings (European Commission 2019). Thus, governments of various countries are outlining strategic plans to significantly increase the use of digital health services (OECD 2023), positioning them as a primary mode of service provision when suitable for the client's situation (Finnish Government 2023).

As healthcare is increasingly relying on technology and digital solutions, the role of registered nurses (hereinafter ‘nurses’) has become evident in the provision of digital services (Isidori et al. 2022). While digital services present promising opportunities, the transition to providing these services introduces challenges for nurses as it substantially reshapes and expands the demands of their roles. Nurses must adapt to new tasks, constant requirements for learning and parallel responsibilities (Kaihlanen et al. 2023). Proficiency in both technical and clinical aspects of care is essential, emphasising the need for nurses to acquire new competencies and receive organisational and collegial support to navigate these digital service environments effectively (Konttila et al. 2019). Recognising the importance of nurses' digital skills is crucial for ensuring the quality of care in digital environments, as nurses themselves acknowledge that their technological proficiency directly impacts the quality of care they provide for their clients digitally (Beauséjour and Hagens 2022).

Despite strategic goals to enhance the role of digital services in healthcare, there is limited understanding of the type and extent of digital client work carried out by nurses and the associating factors. Research in this area is essential for improving nurses' working conditions, supporting organisational support structures and ensuring high‐quality client care.

## 2 Background

The rising costs of healthcare and increasing service demands of the ageing population have underscored the need for widening the methods of care delivery (Penny, Bradford, and Langbecker 2018). Digital services provide opportunities to recognise the clients' needs and deliver healthcare more effectively and sustainably by optimising limited resources (European Commission 2019; World Health Organisation 2021). They have been proposed as a solution to alleviate the international shortage of healthcare professionals (Alshahrani 2022) through improved resource allocation and optimised professional workflows (Socha‐Dietrich 2021). While digital services offer promising opportunities, the transition to providing them presents significant challenges for professionals by substantially changing their work and necessitating adaptation to new tasks and parallel responsibilities, along with reducing the time available for direct contact with clients (Kaihlanen et al. 2023).

As registered nurses comprise the largest group of healthcare professionals and often serve as the initial point of contact for healthcare clients (American Association of Colleges of Nursing 2023), they also have a significant role in delivering digital services to their clients. Nurses have been reported to monitor the client's condition, provide care instructions and information and facilitate communication and interaction using digital methods in client care (Chiang, Wang, and Hsieh 2021; Murphy et al. 2021). Video consultations enable virtual meetings with clients, utilising a webcam or mobile phone camera (Brinker et al. 2018). Additionally, secured messaging enables either immediate synchronous communication (such as live chat) with the client or asynchronous transmission of the client's health data for subsequent review and consultation (Brinker et al. 2018). Clients can also be provided with comprehensive information and support without direct contact by using, for example, a digital care pathway (Haverinen et al. 2024).

It is known that the adoption of digital client work among nurses has significantly increased during the COVID‐19 pandemic (Beauséjour and Hagens 2022; Regragui et al. 2023) and is expected to continue increasing in the future (Fletcher, Read, and D‐Adderio 2023). However, limited information exists on the amount of their digital client work and the specific environments in which it occurs. A Canadian study reported that 27% of nurses used video consultations and 36% used secured messaging in 2020. Digital client work was predominantly done by nurses working in primary or community care environments, such as housing services and home‐based care (Beauséjour and Hagens 2022). However, it is essential to note that this study was performed before the significant increase in digital healthcare services due to the COVID‐19 pandemic.

There are many factors that can influence digital client work among nurses. The successful digital client work is heavily reliant on the clinical experience, and therefore, the use of digital methods in client care is not advisable for early career nurses (Isidori et al. 2022; Konttila et al. 2019). However, an age‐related discrepancy has been previously observed, and nurses working digitally with their clients have been reported to be younger and have fewer years of practice than those not using digital methods in client care (Beauséjour and Hagens 2022). Late‐career nurses often possess advanced experience and nursing skills, and it is crucial for healthcare organisations to foster supportive work environments that not only guarantee equal career opportunities for older nurses but also facilitate the restructuring of their roles to promote their well‐being and sustainability at work (Buchan, Catton, and Shaffer 2020).

The provision of digital services requires nurses to develop new skills beyond technological proficiency, including effective communication with clients in digital channels (Isidori et al. 2022; Jarva et al. 2022), awareness of suitable digital alternatives (Jarva et al. 2022) and minimising information security risks (OECD 2023; Schoenthal and Pouncey 2022). Additionally, nurses need the motivation to efficiently work digitally with clients, and their digital dedication could be associated with the extent of digital client work. Digital dedication is a positive and fulfilling motivational state towards the use of technology at work (Mäkiniemi, Ahola, and Joensuu 2020), and similar to work dedication, it is characterised by enthusiasm, inspiration and pride while concentrating on one's digital tasks (Schaufeli et al. 2002). Motivation and performance in digital client work can be influenced by the social environment, including support from supervisors and the work community (Konttila et al. 2019; Penny, Bradford, and Langbecker 2018; Virtanen et al. 2021).

Despite the significant increase in the provision of digital healthcare services and the strategic goals of expanding their role, there is a lack of comprehensive information regarding the extent of digital client work among nurses and the factors influencing it in various healthcare environments. Previous studies have predominantly focused on the perspectives of professionals and clients regarding specific digital services and have mainly concentrated on physicians' digital client work. Understanding the extent of nurses' digital client work is crucial, as nurses play a key role in multidisciplinary care, often serving as the primary contact for clients and managing the provided digital services. Research on nurses' digital client work is essential for improving their working conditions and helping organisations create stronger support structures, ultimately ensuring high‐quality client care. The results of our study can also serve as a baseline for monitoring the development of digital client work among nurses in the future.

## 3 The Study

### 3.1 Aim

The aim of this study was to describe the frequency of different types of digital client work among registered nurses in Finland. Moreover, we aimed to examine associations of multiple factors related to nurses' individual characteristics and the work environment with (a) digital client work and (b) frequent digital client work.

### 3.2 Design

This survey study employed a cross‐sectional design. To enhance the quality of reporting, we utilised the STROBE checklist specifically tailored for cross‐sectional studies (STROBE 2024).

### 3.3 Setting

Finland is one of the leading countries in digitalisation, where the adoption rate of digital healthcare services among working‐age residents is relatively high (European Commission 2022). An ageing population and the increasing prevalence of chronic conditions (OECD/European Observatory on Health Systems and Policies 2021) have increased the demand for health services and added pressure on healthcare service systems, similar to many other countries. Alongside an uneven geographic distribution of healthcare resources, these have contributed to a concerning imbalance in service accessibility (OECD/European Observatory on Health Systems and Policies 2021). Large‐scale health and social services reform was implemented in Finland at the beginning of 2023, and the responsibility for providing services shifted from the municipalities to the regions (well‐being services counties and the city of Helsinki) (Ministry of Social Affairs and Health 2024). Additionally, the shortage of nurses is severe across the entire country (Ministry of Economic Affairs and Employment 2022). The Finnish government aims to address these challenges by prioritising the use of digital healthcare services, intending to make them the primary mode of service delivery whenever appropriate for the client's situation (Finnish Government 2023).

### 3.4 Participants and Data Collection

The data for this study were collected from registered nurses in Finland over a 2‐week period in April 2023. Because public health nurses, midwives and paramedics are also licensed as registered nurses in Finland (Finnish Nurses Association 2024), they were included in the study. The data collection was a part of the monitoring of digital healthcare and social welfare to evaluate the status of digitalisation. The questionnaire focused on nurses' experiences with the usability of electronic health records and client information systems, as well as their proficiency in using these systems. It also included questions regarding digital client work and the support received from supervisors and colleagues. The questionnaire is available online (Finnish Institute for Health and Welfare 2023).

The survey was developed through extensive collaboration with professionals across various fields. Leading experts from the Finnish Institute for Health and Welfare and the University of Eastern Finland contributed to its creation, and experts from the University of Oulu, the University of Lapland, the Finnish Medical Association and Aalto University were consulted. The pilot version was tested with information management students who had backgrounds as registered nurses, and adjustments were made to individual questions based on their feedback. It was anticipated that responding to the questionnaire would take approximately 20 min.

Two Finnish professional associations, the Union of Health and Social Care Professionals—Tehy and the Finnish Nurses Association, distributed the questionnaires by sending an online link via email to their 18‐ to 65‐year‐old members. The possibility to respond to the questionnaire was offered in Finnish, Swedish and English. A reminder was sent once. A total of 3065 nurses responded to the questionnaire, but 95 responses were excluded because the respondents stated that they did not use electronic health records or client information systems. This exclusion was built into the questionnaire, automatically ending it for those who did not use these systems because the focus of the survey was specifically on the use of information systems.

### 3.5 Ethical Considerations

The research was conducted following good scientific practice and principles of research integrity (Finnish National Board on Research Integrity TENK 2023). Respondents received information about the study that emphasised that their participation in the data collection was completely voluntary. The completion of a questionnaire was interpreted as providing informed consent. Ethics approval for the data collection was obtained from the Institutional Review Board of the Finnish Institute for Health and Welfare (THL/634/6.02.01/2023).

### 3.6 Measures

The items used to measure the extent of different types of digital client work and variables related to individual characteristics and the work environment of the nurses will be presented in the next paragraphs. The variables are presented in greater detail in Data S1.

#### 3.6.1 Dependent Variables

The study used two dependent variables: digital client work and frequent digital client work. First, respondents were asked if they did digital client work, excluding work conducted via telephone. We focused specifically on digital client work, excluding work conducted via telephone, because digital services are increasingly seen as solutions to many contemporary challenges. Unlike telephone services, which have been in use for a long time in Finland, digital services require new skills and roles from professionals.

Subsequently, those who did digital client work were asked about the type and frequency of digital client work during the past 6 months. Different digital service methods were: (1) video consultations, (2) real‐time or non‐real‐time communication with the client, either in anonymous services or services where the client is identified (*secured messaging*) and (3) promoting the client's care in a digital service (*digital promotion of care*) without direct client contact. Response options were: (a) daily, (b) weekly, (c) monthly, (d) less frequently, (e) not at all. For the analyses, the responses were coded as less frequent (response options c and d) and frequent (response options a and b) digital client work.

#### 3.6.2 Independent Variables

The independent variables encompassed factors related to both individual characteristics and the work environment of the nurses.

Factors related to individual characteristics included respondents' *gender* (female, male, other or prefer not to tell) and *career stage* (mid‐career, early career, late career). Due to the low number of respondents who had chosen ‘other’ or preferred not to disclose their gender, these responses were coded as missing to avoid potential bias caused by the large differences in group size. Respondents who had graduated during the past 5 years were regarded as early career nurses, whereas respondents aged 60 years and older were regarded as late career nurses. Other respondents were interpreted to belong to the mid‐career stage. Age was only used as descriptive statistics due to its high correlation with career stage. This approach was chosen because it accounts for the fact that many nurses may transition into nursing later in life, making the number of years since qualification alone less indicative of their remaining career length. By considering both the time since graduation and age, we aimed to better capture this diversity.

Respondents were asked to assess their proficiency in a digital working environment and skills in information security. Response options were (a) excellent, (b) good, (c) satisfactory and (d) poor. Additionally, the option ‘my organization does not require this skill’ was provided. For the analyses, the responses were coded as low (response options c and d) and good (response options a and b). Response option ‘my organization does not require this skill’ was coded as missing.

Digital dedication was evaluated with three items regarding enthusiasm, inspiration and pride in utilising technology in work as part of the validated Techno‐Work Engagement Scale (TechnoWES) (Mäkiniemi, Ahola, and Joensuu 2020). Response options for each of the items were: 1 = daily, 2 = weekly, 3 = monthly, 4 = less frequently and 5 = not at all. For the analyses, the items were reverse‐coded and a mean variable ranging from 1 to 5 was calculated for each respondent to represent the level of their digital dedication. In the analyses, the variable was used as a continuous variable. The Cronbach's alpha value for the items was 0.92, representing excellent internal consistency (Taber 2018).

Factors related to the work environment of the nurses included *working sector* (public, private/third sector) and primary *working unit* (inpatient care, acute care, outpatient care, home‐based care, housing services and others, such as private medical clinic, laboratory and imaging services).

Finally, the respondents who reported digital client work were asked about the amount of *support for digital client work* from (1) their immediate supervisor and (2) their colleagues. The responses were given on a 5‐point scale (1 = very much to 5 = not at all). For the analyses, the items were binary coded as low level (somewhat, only a little, not at all) and high level of support.

### 3.7 Data Analysis

Descriptive statistics were used to summarise the type and frequency of digital client work among nurses and participant characteristics. Binary logistic regression analyses were used to examine the associations of independent variables with (a) digital client work and (b) frequent digital client work among nurses (in separate analyses). First, separate univariable analyses were conducted to examine the association of each independent variable with the dependent variables (Model 1). Second, multivariate models including all the independent variables (career stage, gender, proficiency in digital working environment, information security skills, digital dedication, working sector, primary working unit, support for digital client work from the supervisor and colleagues) were formed (Model 2). Statistical analyses were adjusted for the location of employment due to the unequal distribution of respondents across the well‐being service counties of Finland.

Odds ratios, 95% confidence intervals and *p*‐values are reported. Assumptions of multivariable models (Kirkwood and Sterne 2003) were tested, and there was no multicollinearity in the models. Due to a lack of response to some items, the number of observations varied in the analyses. SPSS 29 was applied to the analyses.

## 4 Results

### 4.1 Characteristics of the Sample

The age of the respondents ranged from 23 to 65 years (mean 47.16 years) and the majority (*n* = 2077, 70.0%) were in the mid‐career stage. Respondents were predominantly females (*n* = 2728, 91.9%) and employed in the public sector (*n* = 2527, 85.3%). Working in inpatient care (*n* = 676, 22.8%) and outpatient care (*n* = 860, 29.0%) were most common compared to other working units. Slightly over half of the respondents (*n* = 1383, 54.3%) reported good proficiency in digital working environments, and the majority (*n* = 2430, 83.6%) reported good information security skills. Respondents' level of digital dedication was moderate. Of the nurses who worked digitally with their clients, half (*n* = 435, 50.3%) reported a high level of support for digital client work from their colleagues, whereas only 14% (*n* = 123) perceived the same level of support from their supervisor. The characteristics of the respondents are presented in Table 1.

**TABLE 1:** The characteristics of the study sample, *n* = 2970.

	In total	Digital client work	Frequent digital client work
	*n* = 2970	*n* = 871	*n* = 599
Individual characteristics			
Age, years, *n* (%)^a^			
< 35	455 (15.3)	120 (13.8)	81 (13.5)
35–44	702 (23.6)	205 (23.5)	144 (24.0)
45–54	927 (31.2)	266 (30.5)	178 (29.7)
55–65	886 (29.8)	280 (32.1)	196 (32.7)
Career stage, *n* (%)			
Mid‐career	2077 (70.0)	615 (70.8)	425 (71.2)
Early career	463 (15.6)	122 (14.0)	84 (14.1)
Late career	426 (14.4)	132 (15.2)	88 (14.7)
Gender *n* (%)			
Female	2728 (91.9)	806 (92.5)	556 (92.8)
Male	214 (7.2)	60 (6.9)	40 (6.7)
Proficiency in digital working environment, *n* (%)			
Low	1166 (45.7)	341 (41.5)	215 (38.0)
Good	1383 (54.3)	480 (58.5)	351 (62.0)
Information security skills, *n* (%)			
Low	477 (16.4)	121 (14.1)	85 (14.4)
Good	2430 (83.6)	737 (85.9)	506 (85.6)
Digital dedication mean, SD^b^	2.92 (1.10)	3.16 (1.13)	3.27 (1.13)
Factors related to work environment			
Working sector, *n* (%)			
Public	2527 (85.3)	728 (84.0)	491 (82.4)
Private/other	434 (14.7)	139 (16.0)	105 (17.6)
Primary working unit, *n* (%)			
Inpatient care	676 (22.8)	92 (10.6)	34 (5.7)
Acute care	507 (17.1)	69 (7.9)	36 (6.0)
Outpatient care	860 (29.0)	467 (53.6)	380 (63.4)
Home‐based care	199 (6.7)	48 (5.5)	21 (3.5)
Housing services	323 (10.9)	50 (5.7)	23 (3.8)
Other	403 (13.6)	145 (16.6)	105 (17.5)
Support to digital client work from supervisor, *n* (%)			
Low level		743 (85.8)	504 (84.3)
High level		123 (14.2)	94 (15.7)
Support to digital client work from colleagues, *n* (%)			
Low level		430 (49.7)	273 (45.7)
High level		435 (50.3)	324 (54.3)

^a^ Only used as a descriptive variable.

^b^ Digital dedication was measured on a scale from 1 to 5, with a higher number indicating greater dedication.

### 4.2 Frequency of Different Types of Digital Client Work Among Nurses

**FIGURE 1:**
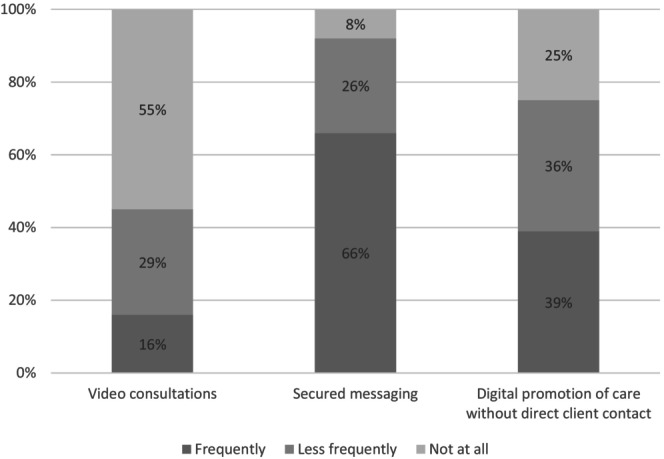
The frequency of different types of digital client work among nurses who reported working digitally with their clients (*n* = 871).

The frequency of different types of digital client work among nurses is presented in Figure 1. A total of 871 nurses (29.6%) reported digital client work during the last 6 months. Only 16% (*n* = 136) of these nurses reported frequent utilisation of video consultations, while 55% (*n* = 462) never used video consultations. Two‐thirds (*n* = 560, 66%) reported that they frequently used secured messaging with their clients. Over one‐third (*n* = 326, 39%) frequently promoted the clients' care without direct client contact. Exclusive digital client work was relatively uncommon, as only 5% (*n* = 41) indicated that their work was performed solely using digital service methods.

### 4.3 Associations of Individual and Work‐Related Factors With Digital Client Work and Its Frequency

The results of the binary logistic regression analyses regarding digital client work among nurses are presented in Table 2.

**TABLE 2:** The results of binary logistic regression analyses for digital client work among nurses (*n* = 2456).

	(Model 1) Univariable analyses^a^			(Model 2) Multivariate analysis^b^		
	OR	95% CI	*p*	OR	95% CI	*p*
Individual characteristics						
Career stage						0.097
Mid‐career	Reference			Reference		
Early career	0.86	0.68–1.08	0.181	0.84	0.64–1.10	0.200
Late career	1.07	0.85–1.34	0.577	1.23	0.94–1.62	0.134
Gender						
Female	Reference			Reference		
Male	0.93	0.69–1.27	0.667	1.40	0.98–2.02	0.068
Proficiency in digital working environment						
Low	Reference			Reference		
Good	1.29	1.09–1.53	0.003	1.15	0.93–1.42	0.215
Information security skills						
Low	Reference			Reference		
Good	1.28	1.02–1.60	0.031	1.16	0.88–1.53	0.310
Digital dedication	1.32	1.23–1.44	< 0.001	1.28	1.17–1.40	< 0.001
Factors related to work						
Working sector						
Public	Reference			Reference		
Private/other	1.16	0.93–1.44	0.190	1.20	0.91–1.59	0.196
Primary working unit						< 0.001
Inpatient care	Reference			Reference		
Acute care	1.01	0.72–1.41	0.978	1.02	0.71–1.47	0.921
Outpatient care	7.59	5.87–9.83	< 0.001	8.13	6.10–10.83	< 0.001
Home‐based care	2.02	1.36–2.99	< 0.001	2.07	1.35–3.16	< 0.001
Housing services	1.16	0.80–1.68	0.438	1.11	0.72–1.72	0.626
Other	3.56	2.64–4.81	< 0.001	3.61	2.60–5.07	< 0.001

*Note:* Odds ratios (ORs), 95% confidence intervals (95% CIs) and *p* values.

^a^ Model included the main effect of each variable.

^b^ Model included all the independent variables simultaneously, adjusted for location of the employment.

Model 2 explained 25% of the variance (Nagelkerke *R* ^2^) in digital client work. In Model 2, digital dedication and the working unit remained statistically significant variables associated with digital client work. Nurses with higher digital dedication had greater odds of digital client work than their less dedicated counterparts. Nurses working in outpatient care, home‐based care and other environments (such as private medical clinics, laboratories and imaging services) had greater odds of digital client work than nurses working in inpatient care.

Table 3 shows the results of binary logistic regression Model 2 for frequent digital client work among nurses. Model 2 explained 26% of the variance (Nagelkerke *R* ^2^) in the frequent digital client work. In Model 2, information security skills, digital dedication, working sector, working unit and support to digital client work from colleagues remained statistically significant variables associated with frequent digital client work. Nurses with good information security skills had lower odds (OR 0.51, 95% CI 0.30–0.87) of frequent digital client work than those with lower skills. Nurses with higher digital dedication had greater odds of frequent digital client work than their less dedicated counterparts. Nurses working in the private or third sector and in outpatient care or other environments had greater odds of frequent digital client work compared to those working in the public sector and in inpatient care. Nurses who received a high level of support for digital client work from their colleagues had greater odds of frequent digital client work than their less‐supported counterparts.

**TABLE 3:** The results of binary logistic regression analyses for frequent digital client work among nurses (*n* = 799).

	(Model 1) Univariable analyses^a^			(Model 2) Multivariate analysis^b^		
	OR	95% CI	*p*	OR	95% CI	*p*
Individual characteristics						
Career stage			0.860			0.752
Mid‐career	Reference			Reference		
Early career	0.99	0.65–1.50	0.956	0.82	0.50–1.34	0.453
Late career	0.89	0.60–1.33	0.584	1.00	0.62–1.63	0.986
Gender						
Female	Reference			Reference		
Male	0.90	0.52–1.57	0.709	1.26	0.65–2.42	0.495
Proficiency in digital working environment						
Low	Reference			Reference		
Good	1.60	1.18–2.15	0.002	1.41	0.96–2.08	0.078
Information security skills						
Low	Reference			Reference		
Good	0.93	0.61–1.41	0.726	0.51	0.30–0.87	0.014
Digital dedication	1.33	1.17–1.52	< 0.001	1.30	1.10–1.53	0.002
Factors related to work						
Working sector						
Public	Reference			Reference		
Private/other	1.49	0.98–2.26	0.060	1.98	1.16–3.38	0.012
Primary working unit			< 0.001			< 0.001
Inpatient care	Reference			Reference		
Acute care	1.86	0.99–3.51	0.055	2.00	0.96–4.18	0.065
Outpatient care	7.45	4.60–12.08	< 0.001	8.63	4.85–15.34	< 0.001
Home‐based care	1.33	0.65–2.70	0.435	1.36	0.60–3.06	0.459
Housing services	1.45	0.72–2.92	0.295	1.57	0.67–3.69	0.298
Other	4.48	2.56–7.83	< 0.001	4.10	2.11–7.96	< 0.001
Support to digital client work from supervisor						
Low level	Reference			Reference		
High level	1.54	0.99–2.40	0.058	1.03	0.59–1.81	0.910
Support to digital client work from colleagues						
Low level	Reference			Reference		
High level	1.68	1.25–2.25	< 0.001	1.73	1.20–2.48	0.003

*Note:* Odds ratios (ORs), 95% confidence intervals (95% CIs) and *p* values.

^a^ Model included the main effect of each variable.

^b^ Model included all the independent variables simultaneously, adjusted for location of the workplace.

## 5 Discussion

The aim of this cross‐sectional survey study was to describe the frequency of different types of digital client work among nurses and examine its associating factors in Finland. In our study, one‐third of the nurses reported working digitally with their clients. The majority of those working digitally with their clients used digital service methods on a daily or weekly basis. Secured messaging emerged as the most frequently used digital service method, while video consultations were used relatively less often. Digital client work was done in varying degrees in different healthcare environments. Of the nurses' individual characteristics, higher digital dedication was associated with both digital client work and frequent digital client work, and lower skills in information security were associated with frequent digital client work. Of the factors related to the nurses' working environment, the working unit was associated with both digital client work and frequent digital client work and the working sector and higher level of support for digital client work from colleagues were associated with frequent digital client work.

Nurses' digital client work in Finland appears to be relatively limited, as only approximately one‐third of nurses reported that they worked digitally with their clients. Given the high expectations set for digital health services and the increasing reliance on their use as the primary method of health service provision (Finnish Government 2023), the extent appears relatively small. Notably, previous research in Canada indicated that 27% of nurses were utilising video conferencing and 36% secured messaging in 2020, just before the outbreak of the COVID‐19 pandemic (Beauséjour and Hagens 2022). However, another Canadian study reported minimal use of video consultations among nurses in primary care both before and during the pandemic. The low utilisation was explained by insufficient technological resources and infrastructure, as well as a lack of adequate training and supervision for nurses (Regragui et al. 2023).

Based on our research findings, it seems that many nurses who work digitally with their clients also have a considerable amount of traditional face‐to‐face interaction with clients. In previous research, nurses have expressed positive perceptions regarding a hybrid model that combines digital and traditional services (Jarva et al. 2022; Regragui et al. 2023). While the hybrid approach is valued by nurses, it is noteworthy that the emerging digital client work rarely replaces traditional care models, but they rather tend to be implemented simultaneously, thereby augmenting the workload of professionals (Kaihlanen et al. 2023). It is crucial for organisations to develop strategies for seamlessly integrating digital client work into nursing roles, creating a meaningful array of tasks for nurses that align with the organisation's objectives. The fragmented implementation of digital healthcare services has been reported as impeding the workflow of professionals (Eloranta et al. 2023), presenting specific challenges for nurses who work digitally alongside physical interactions with the clients (Entezarjou et al. 2020). A study conducted in home‐based care demonstrated that centralising the organisation of digital client work can alleviate professionals' workloads and clarify and enhance their task allocation (Eloranta et al. 2023).

The frequency of video consultations appeared relatively limited among nurses in our study, as more than half of those working digitally with their clients were not involved in any video consultation roles at all. This finding aligns with prior research that observed an infrequent use of video consultations among nurses (Murphy et al. 2021; Regragui et al. 2023). Healthcare professionals have expressed concerns regarding the reduced opportunity to obtain information and the loss of certain nuances in communication while messaging with clients (Entezarjou et al. 2020). Conversely, video consultations have been found to promote more comprehensive interactions with clients, enabling professionals to gather both verbal and visual information, thus potentially reducing communication errors and expediting decision‐making (Penny, Bradford, and Langbecker 2018). On the other hand, asynchronous digital services, such as secured messaging, offer increased flexibility and the opportunity to seamlessly integrate digital client work into professionals' daily workflow (Brinker et al. 2018). By combining the strengths of both video consultations and asynchronous digital services, nurses could effectively manage their responsibilities and provide comprehensive care to clients.

According to our results, those with lower self‐perceived information security skills reported more frequent digital client work than those with good skills. This might suggest that nurses who extensively work digitally with their clients may possess a heightened awareness of potential information security threats, leading them to be more critical of their own information security skills. However, our study design was cross‐sectional. Thus, we cannot draw any conclusions regarding the causality of this association. A recent study observed that while a majority of nurses demonstrated a good level of information security awareness and behaviour, they had less favourable attitudes towards information security. The shift towards digital service delivery has expanded the use of digital platforms, thereby increasing potential vulnerabilities and underscoring the importance of information security skills among professionals (World Health Organisation 2021). Additionally, frequent digital client work may create opportunities for remote work, increasing the risk of threats as information systems are accessed beyond hospital networks, and family members might overhear confidential client information (Schoenthal and Pouncey 2022). Due to the dynamic nature of technology and evolving information security threats, continuous education and training in information security are essential for nurses.

Our findings suggest that nurses' proficiency in a digital working environment is not associated with digital client work. In our study, nurses with higher skills did not work digitally with their clients more frequently than those with lower skills, and those with frequent digital client work did not necessarily report better proficiency. However, it could be assumed that skills improve with increased digital client work. This underscores the importance of continuous education and training in digital competence at every stage of a nurse's career. Given that the career stage of nurses was not associated with digital client work and that nurses at the beginning of their careers also work digitally with their clients, it becomes crucial to integrate comprehensive digital competency training into their educational curricula (Fletcher, Read, and D‐Adderio 2023).

Nurses with a higher level of digital dedication had a greater likelihood for digital client work and more frequent digital client work compared to their less dedicated counterparts in our study. Digitally dedicated nurses may be more willing than others to apply for job roles that offer the opportunity to work digitally with clients. However, the association may be reciprocal, and digital client work might also enhance nurses' digital dedication as they become more familiar with digital technologies and potentially perceive more benefits in their use. Organisational efforts should be made to foster a workplace atmosphere that is conducive to digitalisation as the social environment influences professionals' motivation to utilise technologies in their work and their attitudes towards digitalisation (Konttila et al. 2019). Further, dedication has been observed to be associated with professionals' better performance and commitment at work (Bakker and Demerouti 2007). The motivation of professionals has been identified as a necessary component in promoting their competence and gaining long‐term benefits from the use of digital health technology (Virtanen et al. 2021).

According to our results, nurses work digitally with their clients to varying degrees in different healthcare environments. Nurses working in the private or third sector were more likely to work digitally with their clients than those working in the public sector. One possible explanation for this disparity could be the distinct client populations served in these sectors. In Finland, the private sector primarily encompasses general practice and specialised outpatient care, whereas specialised medical and emergency care predominantly take place in public sector hospitals, which constitute the majority of hospitals in the country (Ministry of Social Affairs and Health 2024). Our results also indicate that nurses working in outpatient units, home‐based healthcare services and other environments were more likely to work digitally with their clients and reported higher frequency of digital client work than those working in inpatient care. Inpatient units provide care for clients who require monitoring and repeated or continual treatment and often deal with serious illness or trauma, making digital service methods less appropriate. Further research and organisational considerations are needed to explore whether the amount of digital client work among nurses could be increased in working environments that currently have lower utilisation. In home‐based healthcare services, for example, monitoring the client's health status and well‐being, tracking medication intake and nutritional status (Eloranta et al. 2023) and communication (Chiang, Wang, and Hsieh 2021) could be effectively done using digital service methods.

Our results indicate that support received from colleagues for digital client work may hold more significance for nurses than support received from supervisors. Nurses who perceived receiving a high level of support from their colleagues demonstrated a higher tendency to frequent digital client work than those who felt less supported. Interestingly, this result persists despite a significantly larger number of nurses reporting substantial support from colleagues compared to supervisors. The social nature of nursing, where teamwork is important and professionals are strongly influenced by their colleagues (Du Toit 1995), underscores the pivotal role of colleague support in digital client work. A previous study emphasises the importance of collegial support among healthcare professionals as it affects teamwork climate and values, which influence the adoption of new technology (Rippen et al. 2013). More organisational efforts should focus on improving opportunities for colleagues to support each other in digital client work. As feedback is an important aspect of social support (Katz 1995), its provision should be emphasised. Although support from a supervisor was not associated with digital client work according to our results, considering the limited number of nurses perceiving substantial supervisor support, supervisors require additional education and resources for supporting professionals transitioning to digital service methods.

### 5.1 Limitations

There are some limitations in our study that need to be considered when interpreting the results. Because the survey was distributed through professional associations, the response rate and the exact representativeness of the data cannot be determined. Additionally, a power analysis was not conducted to establish the ideal number of respondents, which can be seen as a limitation. However, the large number of respondents and the variation in their demographic characteristics suggest that the sample is a good representation of the target group (Finnish Nurses Association 2021) and is sufficient for reliable analysis.

Our findings are based on self‐reported data, which could lead to problems associated with the common method variance and the inflation of the strength of relationships. Although multiple factors were adjusted for in the analyses, the possibility of residual confounding still remains, such as the nurses' ability to influence the amount of their digital client work. Moreover, cross‐sectional survey data do not allow us to draw any confirmatory causal inferences from the results.

The concept of digital client work was not comprehensively defined in the survey questionnaire, potentially leading to ambiguity in the responses. For instance, some respondents might have interpreted digital client work to exclusively involve synchronous remote consultation work, overlooking other aspects related to digital client work. Additionally, the question about respondents' skills in digital working environments may have been unclear and inadequately described. Furthermore, the inclusion of various digital services in different studies complicates the comparison of the extent and associated factors of digital client work across research. This variation underscores the need for standardised definitions to enable more accurate comparisons and a better understanding of digital client work's impact in various contexts.

In the questions addressing skills in information security and digital working environments, the response option ‘my organization does not require this skill’ may have introduced response bias and could reflect more about the respondent's personal attitude rather than the actual skills required by the organisation. It will be important to reconsider the justification for including this response option in future surveys.

Additionally, as well‐being services counties became responsible for organising healthcare and social welfare services in Finland at the beginning of 2023 (Ministry of Social Affairs and Health 2023), not all nurses were reached by the professional associations due to the change of employer's name and email addresses. Consequently, the number of participating nurses in the study was smaller than in previous national surveys that monitored and evaluated healthcare and social welfare information systems from the nurses' perspective (Finnish Institute for Health and Welfare 2023). Variation was observed in the geographical distribution of responses. This was accounted for in the analyses by adjusting for workplace location. Due to these limitations, the proportion of nurses working digitally with their clients may be greater than our study results suggest.

## 6 Conclusions

The findings of this cross‐sectional study showed that about one‐third of registered nurses worked digitally with their clients in Finland. Further organisational considerations are essential to evaluate whether the volume of digital client work could be increased in environments where it is currently infrequent. Particularly, the expansion of video consultations should be comprehensively deliberated upon. To meet the high expectations set for digital services, it is crucial to ensure adequate resourcing, enabling clients to seamlessly access these services.

Considering that digital environments are reshaping the traditional responsibilities of nurses and transforming their professional roles, organisational efforts should be made to foster a workplace atmosphere that is conducive to digitalisation and enhances the dedication of nurses to digital client work. Continuous education and training in information security are essential for nurses to safeguard the clients' sensitive health information. Initiatives should concentrate on enhancing opportunities for colleague support in digital client work, emphasising the importance of feedback as a crucial component of social support. Moreover, supervisors need additional education and resources to support professionals' effective utilisation of digital service methods and to foster digitalisation within the nursing profession.

## Author Contributions

E.K. and T.V. made substantial contributions to conception and design, or acquisition of data, or analysis and interpretation of data. E.K., A.‐M.K., L.V., T.V. and T.H. were involved in drafting the manuscript or revising it critically for important intellectual content. E.K., A.‐M.K., L.V., T.V. and T.H. gave final approval of the version to be published. E.K., A.‐M.K., L.V., T.V. and T.H. agreed to be accountable for all aspects of the work in ensuring that questions related to the accuracy or integrity of any part of the work are appropriately investigated and resolved.

## Ethics Statement

The study was conducted in accordance with the Declaration of Helsinki and following good scientific practice and principles of research integrity. Ethics approval for the data collection was obtained from the Institutional Review Board of the Finnish Institute for Health and Welfare (THL/634/6.02.01/2023). Respondents were provided with information about the study and participation in data collection was completely voluntary. The completion of a questionnaire was considered as giving informed consent.

## Conflicts of Interest

The authors declare no conflicts of interest.

## Supporting information


Data S1.

## Data Availability

The data that support the findings of this study are available on request from the corresponding author. The data are not publicly available due to privacy or ethical restrictions.
