# Supplementary material for: Registered Nurses' Digital Client Work and Associating Factors: A Cross‐Sectional Study
Source: J Adv Nurs. 2024 Sep 28;81(7):3703–14. doi: 10.1111/jan.16485 (PMC12159359; doi:10.1111/jan.16485)
Supplement: Supplementary file 1 — Data S1. [file JAN-81-3703-s001.docx]

Supplementary file 1.

### The used variables

**Digital client work**

Does your work involve digital client/patient contact? This does not refer to work carried out by telephone.

[_] yes [_] no

**Frequent digital client work**

How often has your work included the following digital service events in the past 6 months?

**Client video reception**

[_] daily [_] weekly [_] monthly [_] less frequently [_] not at all

**Real-time digital communication with the client**

[_] daily [_] weekly [_] monthly [_] less frequently [_] not at all

**Non-real-time digital communication with the client**

[_] daily [_] weekly [_] monthly [_] less frequently [_] not at all

**Helping the client’s care in a digital service without direct client**

[_] daily [_] weekly [_] monthly [_] less frequently [_] not at all

**Proficiency in a digital working environment and skills in information security**

How well do you feel you master the following skills required by information systems?

**Working in a digital healthcare environment**

[_] excellent [_] good [_] satisfactory [_] poor [_] my organization does not require this skill

**Complying with data protection and data security principles in daily work**

[_] excellent [_] good [_] satisfactory [_] poor [_] my organization does not require this skill

**Digital dedication**

How often do you have the following kinds of feelings and thoughts?

**I am enthusiastic about utilising technology in my job**

[_] daily [_] weekly [_] monthly [_] less frequently [_] not at all

**Utilising technology inspires me in my job**

[_] daily [_] weekly [_] monthly [_] less frequently [_] not at all

**I am proud that I utilise technology in my work**

[_] daily [_] weekly [_] monthly [_] less frequently [_] not at all

*working sector*

**Working sector**

Which sector pays your salary?

[_] public sector [_] private sector [_] third sector

**Primary working unit**

*Where do you work more specifically?*

*Hospital*

*[_] Inpatient ward (e.g. internal diseases)*

*[_] Emergency clinic*

*[_] Intensive care or monitoring unit*

*[_] Operating room*

*[_] Delivery room*

*[_] Outpatient clinic*

*[_] Other hospital unit*

*Public health and social services centre/ health centre*

*[_] Patient/ client appointment*

*[_] Inpatient ward*

*[_] Emergency clinic*

*[_] Maternity health clinic or child health clinic*

*[_] Other social and health centre unit*

*Other environments*

*[_] Private medical clinic or centre*

*[_] Occupational health*

*[_] Emergency care or ambulance*

*[_] Home nursing*

*[_] Hospital-at-home*

*[_] Service housing, supported housing or housing services (e.g. for older people, persons with intellectual disabilities or mental health and substance abuse rehabilitation)*

*[_] Service housing (with 24/7 assistance)*

*[_] Institutional care (e.g. for persons intellectual disabilities, child welfare clients or persons with substance abuse problems)*

*[_] Research unit (e.g. laboratory, imaging services)*

*[_] School or student health care*

*[_] Other*

***Support for digital client work***

Do you feel that you have received enough support for doing digital work?

**from my immediate supervisor**

[_] very much [_] fairly much [_] somewhat [_] only a little [_] not at all

**from my colleagues**

[_] very much [_] fairly much [_] somewhat [_] only a little [_] not at all
